# The Rapid Perceptual Impact of Emotional Distractors

**DOI:** 10.1371/journal.pone.0129320

**Published:** 2015-06-15

**Authors:** Briana L. Kennedy, Steven B. Most

**Affiliations:** School of Psychology, University of New South Wales, Sydney, NSW, Australia; University of Groningen, NETHERLANDS

## Abstract

The brief presentation of an emotional distractor can temporarily impair perception of a subsequent, rapidly presented target, an effect known as *emotion-induced blindness* (EIB). How rapidly does this impairment unfold? To probe this question, we examined EIB for targets that immediately succeeded (“lag-1”) emotional distractors in a rapid stream of items relative to EIB for targets at later serial positions. Experiments 1 and 2 suggested that emotional distractors interfere with items presented very soon after them, with impaired target perception emerging as early as lag-1. Experiment 3 included an exploratory examination of individual differences, which suggested that EIB onsets more rapidly among participants scoring high in measures linked to negative affect.

## Introduction

Emotionally powerful distractors can spontaneously impair awareness of closely following targets in a rapid stream of stimuli–a phenomenon known as *emotion-induced blindness* (EIB) [1–6]. In EIB experiments, emotional distractors–both negative and positive [2]–spontaneously impair target accuracy relative to when the targets appear after emotionally “neutral” distractors.

In the context of previously documented effects, EIB seems most closely to resemble the *attentional blink (AB)*, a robust perceptual phenomenon where people fail to see the second of two rapidly presented targets (T2) if it follows too closely in the wake of a first target (T1) [7,8]. Emotional stimuli can affect the patterns observed in the AB: they tend to increase the AB when serving as T1 [9–11] and decrease the AB when serving as T2 [9–13]. Some neural evidence is consistent with the notion that EIB and the AB are mechanistically similar. For example, in one EIB study that employed event-related potentials, the effect went hand-in-hand with target-distractor trade-offs in the amplitude of the N2 and P3 components [14], and similar trade-offs have been found between T1 and T2 in a standard attentional blink task [15,16].

However, despite similarity between EIB and the AB, there have been suggestions that the two phenomena involve different mechanisms and that EIB needs to be understood as a phenomenon in and of itself [6,17]. Not only does EIB occur spontaneously whereas the AB relies on purposeful attention to a first target, but EIB also appears to be spatially localized while the AB appears not to be [17,18]. Indeed, even at the neural level, evidence is mixed. Studies show that the N400—a component linked with semantic processing–was sensitive to stimuli appearing as “blinked” T2s in an AB task [15,19], suggesting that the AB stems from a relatively late-stage bottleneck. Such a bottleneck would seem to be inconsistent with what appears to be the spatially localized nature of EIB [6,17]. In studies using fMRI, the AB following a non-emotional T1 was associated with activation in the right intra-parietal and frontal cortex and in the anterior cingulate cortex [20], whereas a prolonged AB following an emotional (task relevant) target was associated with activation in orbitofrontal cortex, anterior cingulate cortex, and insula [10]. Thus, there is evidence that EIB and the AB may share some neural drivers but not others. With such mixed evidence, the more we learn about the characteristics of EIB, the more we will know whether its mechanisms can be appropriately understood through the lens of the AB literature. *Lag-1-sparing*–described below–is a common and informative characteristic of the AB, which lends heavily to theories about the phenomenon. Here, we also seek to understand performance at lag-1 in EIB in order to gain further insight into its underlying properties.

In the AB, *lag-1-sparing* refers to the frequent observation that perception of T2 remains relatively unimpaired when it is the item immediately succeeding T1 [7,21–23], an observation that has influenced many models of the attentional blink [7,21,22,24]. For example, one model posits a sluggish closing of an attentional “selection gate” following T1, which enables an immediately subsequent T2 to be processed before the initiation of temporary attentional suppression [7,8,25]. Additionally, proponents of a “temporary loss of control” account of the AB have suggested that the first item following T1 disrupts and reconfigures an attentional filter, which induces the AB but also enables lag-1-sparing, as there would be no intervening, reconfiguring item between T1 and T2 [21,22,26,27].

Although studies of EIB have probed target perception at various lags following an emotional distractor [28], most experiments have probed the second serial position following the distractor (“lag-2”) at the earliest. (EIB experiments have mostly presented stimuli at a rate of 100-ms/item, so that the onset of targets at lag-2 occurs 200-ms after the onset of the emotional distractor.) In one exception, an emotion-induced blindness task probed the first post-distractor serial position and found impaired performance for items at lag-1 following emotional distractors (although individual differences in attentional control did predict the difference between the emotional and non-emotional conditions at lag-1) [29]. Notably, however, that study did not find robust EIB when collapsed across participants, which the authors suggested might have been due to the relative mildness of the study’s emotional stimuli (faces with emotional expressions). Another possible reason that EIB did not emerge robustly in that study is that the target and distractors were each defined by a feature singleton (i.e., a unique color amidst grayscale images), so both neutral and emotional distractors might have been prone to capture attention had participants actively sought a target defined by a feature singleton [30]. Thus, while this earlier study is relevant to the goals of the current study, the degree to which its findings can be assumed to generalize to EIB broadly is complicated by a combination of factors. In a second exception that employed emotionally powerful scene images, lag-1 was probed but performance was not directly compared to performance at later lags within the same experiment [3], leaving open the possibility that target perception at lag-1 is generally more accurate than at lag-2.

Theories of the AB may contribute to understanding of EIB: one possibility is that EIB engages the same mechanisms as the AB, in which case, target detection should be intact at lag-1 in EIB. Another reason to suspect strong target performance at lag-1 is that it may take some time for individuals to extract emotional value from a rapidly presented image, leading to a brief delay before an emotional stimulus begins to compete with subsequent items. Indeed, ERP research has identified characteristic neural signatures that onset about 150-ms after stimulus presentation [31–33], as well as evidence that emotional stimuli suppress neural responses linked to subsequent stimuli about 200-ms after emotional stimulus onset [31,14]. Although these effects occur quickly, their timing does provide enough of a window for a target to be perceived at lag-1 prior to the onset of spatiotemporal competition. Thus, the presence of lag-1-sparing in the current set of experiments could be interpreted as consistent either with the suggestion that EIB engages the same mechanisms as the AB or with a (delayed) perceptual competition account.

In order to further triangulate the results, Experiment 3 incorporated exploratory self-report measures of attentional control and trait negative affect, as affect-related individual differences might correlate with the speed with which people register the emotionality of a stimulus. This prediction stemmed from previous findings that emotional stimuli are processed faster than less emotional stimuli [34]. The rate at which people extract emotional information may vary as a function of affect-related individual differences, such that extraction of emotional information from a visual stimulus takes long enough to leave perception of immediately subsequent targets intact in some people but not others. In contrast, if the possible presence of lag-1-sparing in EIB reflects the type of fundamental attention mechanisms implicated in the attentional blink, there is less reason to predict that it should vary systematically with trait negative affect, although trait negative affect might be predicted to modulate the amplitude of EIB. Consistent with the possibility that trait affect modulates the amplitude of EIB, trait [35–37] and state [38–39] affect have previously been found to modulate the magnitude of non-emotional versions of the AB. For example, individuals with greater dispositional negative affect showed a greater magnitude in AB, while those with greater positive affect demonstrated a smaller AB [35].

In short, performance at lag-1, relative to later lags, should contribute to a better understanding of EIB, considered both on its own and in relation to existing accounts of the phenomenally similar AB. In the present set of experiments, we assessed EIB at lag-1 (100-ms after the onset of an emotional distractor) relative to EIB at later serial positions. In Experiment 1, emotion-induced blindness at lag-1 was specifically compared with emotion-induced blindness at the very next serial position.

## Experiment 1

### Method

#### Participants

Forty-two undergraduates from the University of Delaware (mean age = 19.5 years; 28 female, 14 male) participated in exchange for course credit. We predicted no difference between lag-1 and lag-2 and therefore chose a relatively large sample size to be confident in any null effect. Previous emotion-induced blindness studies have suggested that the recovery in performance from lag-2 to lag-8 represents a large effect [1]. Therefore, if lag-1 performance is poor, the effect should also be large or medium in size, especially compared to the general lack of impairment altogether at lag-1 in attentional blink studies [7]. Thus, we chose a sample size to provide sufficient power to explore lag-1 performance, whether intact or impaired from the emotional distractors. All participants provided written informed consent as approved by the University of Delaware’s Human Subjects Review Board, Approval Number 136021–4. Data from two participants (1 female and 1 male) were excluded from analyses due to failure to record data. All participants were naïve to the purpose of the experiment.

#### Materials and Procedure

Stimuli were presented and responses gathered via the Psychophysics Toolbox for Matlab [40–41]. Stimuli appeared on a 100-Hz CRT monitor measuring 15.2 cm wide x 11.4 cm high. Stimuli were 492 colored, 320x240 pixel photographs. Eighty-four of the images served as the “targets,” which were 42 landscape and architectural images rotated 90 degrees clockwise and counterclockwise. Another 168 images served as the “distractors” comprising of 52 negative, 52 neutral, and 52 scrambled images. Negative distractors were used as the emotional stimuli, consistent with most existing emotion-induced blindness experiments [1–6]. Negative and neutral images were chosen based on ratings of valence and arousal and were mostly gathered from the International Affective Picture System (IAPS) [42], with the rest supplemented by similar images taken from publicly available sources. Stimuli used in this experiment were from the same collection as used in previous emotion-induced blindness experiments in our lab [1], and negative and neutral images were previously rated by a separate group of participants (as cited in [5]). In the rating task, 12 participants rated the distractor images on dimensions of valence (1 = negative, 9 = positive) and arousal (1 = low, 9 = high). The negative and neutral distractors differed significantly for both valence (negative: *M* = 1.73, *SD* = 0.54; neutral: *M* = 4.99, *SD* = 0.45) and arousal (negative: *M* = 6.04, *SD* = 0.69; neutral: *M* = 3.18, *SD* = 0.55) measures (ps<0.001). Scrambled images were the same 52 negative images, each segmented into an 8x6 grid, with the segments randomly rearranged. These served as a control for low-level properties (e.g., color and luminance) of the negative images. The remaining 252 photographs were upright landscape and architectural images that served as filler images.

The experiment included three self-contained blocks and a total of 216 trials (72 trials per block). Each trial consisted of a rapid serial visual presentation (RSVP) of 17 images presented at a rate of 100 ms/item. The stream of images was presented in the center of the screen against a gray background. Depending on the trial, the distractor appeared at serial position 4, 6, 8, 10, or 12, and the target appeared either one position (lag-1) or two positions (lag-2) after the distractor. After every trial, participants indicated, via button press, whether the target was rotated clockwise or counterclockwise. Participants heard a bell through headphones if they answered correctly but heard nothing if they answered incorrectly.

Before beginning the experiment, participants were shown examples of emotional and neutral images used in the experiment to ensure informed consent. They then engaged in a 16-trial practice session, with RSVP rates starting at 200-ms and slowly increasing to the experiment presentation rate of 100-ms. The practice session did not include distractors. Participants were debriefed after the experiment.

### Results

**Fig 1:**
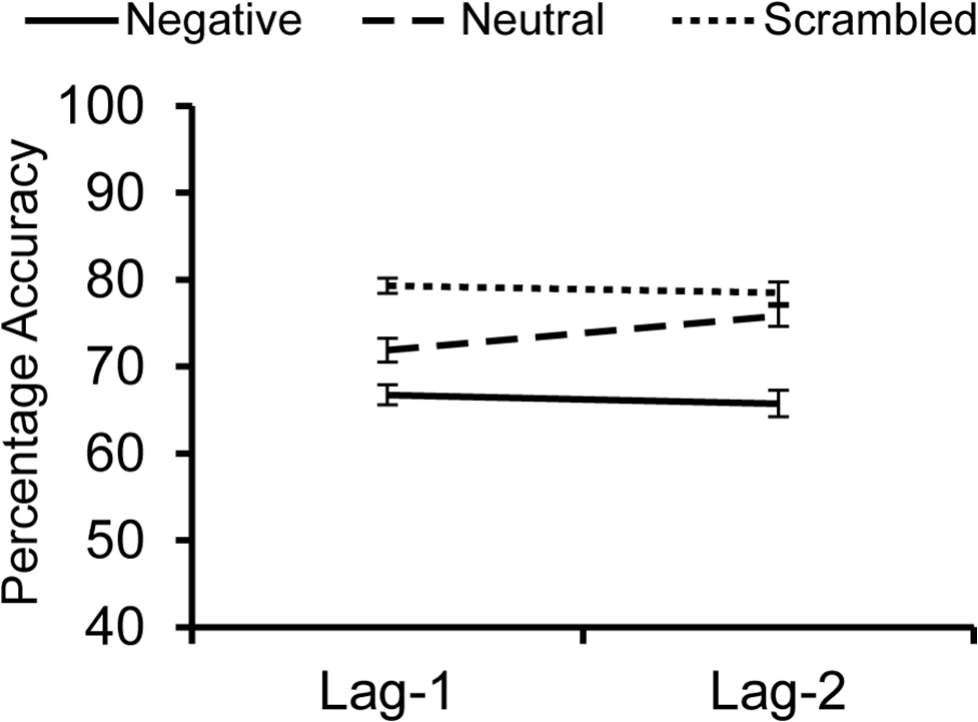
Average percent accuracies for target detection in Experiment 1. Targets were presented one image (lag-1) or two images (lag-2) after negative, neutral, or scrambled distractors. Error bars represent standard error. See text for details.

Percentage accuracy in reporting target rotation served as the primary measure of interest. An overall 3 (Distractor Type: negative, neutral, scrambled) x 2 (lag-1 vs. lag-2) within-subjects ANOVA revealed a significant main effect of Distractor Type, *F*(2,39) = 41.767, *p* < .001, η_p_^2^ = 0.517, with the poorest performance in trials with emotional distractors (lag-1: *M* = 66.7%, *SD* = 13.6%; lag-2: *M* = 65.9%, *SD* = 13.1%*)*, better performance for those with neutral stimuli (lag-1: *M* = 71.7%, *SD* = 12.3%; lag-2: *M* = 76.0%, *SD* = 15.2%), and the best performance in trials with scrambled stimuli (lag-1: *M* = 79.2%, *SD* = 14.5%; lag-2: *M* = 78.7%, *SD* = 18.4%; see Fig 1). There was no significant main effect of Lag, *F*(1,39) = 0.544, *p* = .465, η_p_^2^ = 0.014, indicating that there was no overall difference in performance between lag-1 and lag-2.

The Distractor Type X Lag interaction was marginally significant, *F*(2,39) = 3.095, *p* = .051, η_p_^2^ = 0.074. Follow-up tests revealed that this difference was largely driven by the lag difference in the neutral distractor condition, *t*(39) = 2.002, *p* = .052, *d*_*z*_ = .466. There was no significant difference between lag-1 and lag-2 accuracy in the negative condition, *t*(39) = 0.422, *p* = .675, *d*_*z*_ = .067, or scrambled condition, *t*(39) = 0.392, *p* = .697, *d*_*z*_ = .062. Indeed, a subsequent 2 (Distractor Type: negative vs. neutral) X 2 (lag-1 vs lag-2) ANOVA revealed–in addition to a main effect of Distractor Type (*F*(1,39) = 27.668, *p*<.001, η_p_^2^ = .415)–a significant Distractor Type X Lag interaction, *F*(1,39) = 4.211, *p* = .047, η_p_^2^ = .097, suggesting that recovery following a neutral distractor was faster than recovery following a negative one.

Interestingly, although no evidence of lag-1-sparing following an emotional distractor emerged in the group analysis, roughly half of the participants showed a trend towards better performance at lag-1 than at lag-2. In this experiment, we did not collect supplementary measures that might have helped to reveal systematic individual differences in this regard. However, we follow up on this observation via exploratory, supplemental measures in Experiment 3.

### Discussion

In Experiment 1, detection of targets after emotional distractors was similarly impaired across lag-1 and lag-2. This suggests that there was impairment from the emotional distractors as early as 100ms following its presentation.

However, it remains possible that the negative distractors produced an overall decrement in performance, such that the absence of a difference between lag-1 and lag-2 reflected an impairment that persisted across all lags, which would implicate memorial or response processes rather than perceptual competition. Previous emotion-induced blindness findings suggest that emotion-induced blindness disappears by lag-8 [1]; thus, in Experiment 2 we aimed to rule out a lag-independent account by comparing EIB at lag-1 to EIB at lag-8. If the findings of Experiment 1 reflect an overall decrement unrelated to perceptual processing, then performance at these lags should be roughly equivalent.

## Experiment 2

### Method

#### Participants

Fifteen undergraduates from the University of Delaware participated in Experiment 2 in exchange for course credit (mean age = 19.4 years; 9 female, 6 male). In this experiment, we expected a large difference in performance at lag-1 and lag-8 and therefore chose a sample size commensurate with previous emotion-induced blindness experiments [1]. All participants provided written informed consent as approved by the University of Delaware’s Human Subjects Review Board, Approval Number 136021–4.

#### Materials and Procedure

Experiment 2 used the same materials and procedure as those used in Experiment 1, with the exception that lag-2 trials were replaced with lag-8 trials, and the distractor could instead appear at serial position 4, 5, 6 or 7.

### Results

**Fig 2:**
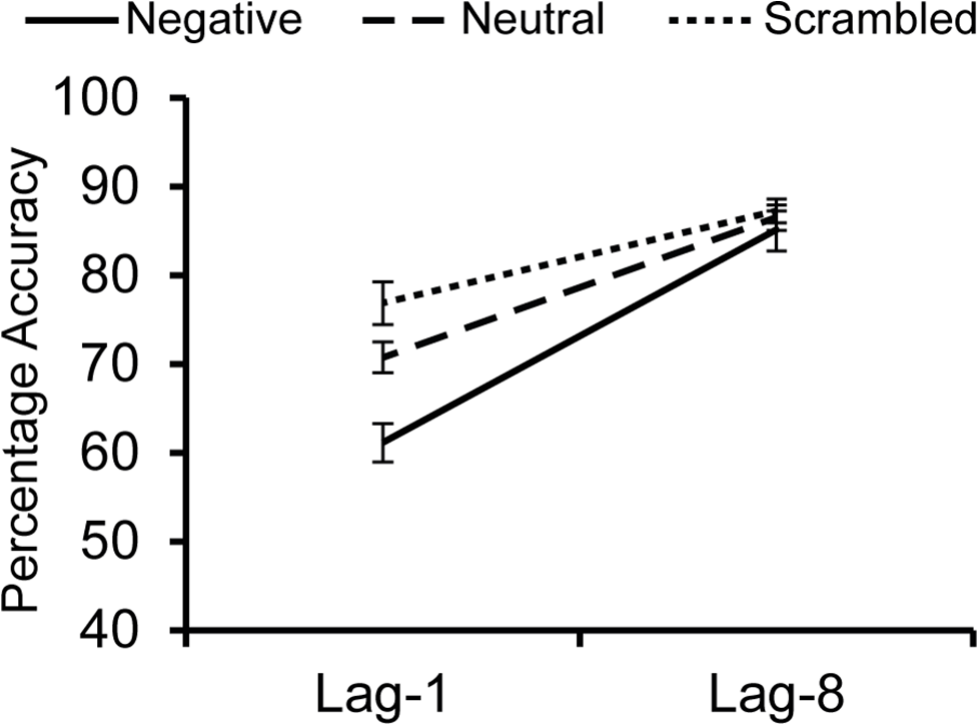
Average percent accuracies for target detection in Experiment 2. Emotion-induced blindness occurred when the target was presented one image (lag-1) after a negative distractor, but not when presented eight images (lag-8) after the distractor.

An overall 3 (Distractor Type: negative, neutral, scrambled) x 2 (lag-1 vs. lag-8) within-subjects ANOVA revealed a significant main effect of Distractor Type, *F*(2, 14) = 7.715, *p* = .002, η_p_^2^ = .355, with the negative distractors inducing the most impairment (lag-1: *M* = 61.1%, *SD* = 12.3%; lag-8: *M* = 85.0%, *SD* = 15.0%), followed by the neutral distractors (lag-1: *M* = 70.7%, *SD* = 10.1%; lag-8: *M* = 86.5%, *SD* = 11.5%), and least impairment following the scrambled (lag-1: *M* = 76.9%, *SD* = 15.5%; lag-8: *M* = 87.2%, *SD* = 11.6%; see Fig 2). The main effect of Lag was also significant, *F*(1,14) = 94.5, *p* < .001, η_p_^2^ = .871, with greater impairment at lag-1 than at lag-8. The Distractor Type X Lag interaction was significant, *F*(2,14) = 6.093, *p* = .006, η_p_^2^ = .303.

Consistent with Experiment 1, in a separate ANOVA there was a main effect of Distractor Type at lag-1, *F*(2,28) = 10.993, *p* < .001, η_p_^2^ = .440. Lag-1 accuracy was significantly impaired in trials with negative distractors compared to those with neutral distractors, *t*(14) = 3.790, *p* = .002, *d*_*z*_ = .978, or scrambled distractors, *t*(14) = 3.807, *p* = .002, *d*_*z*_ = .983. However, there was no significant difference in performance across distractor conditions at lag-8, *F*(2,28) = 0.377, *p* = .690, η_p_^2^ = .026.

### Discussion

Data from Experiment 2 support the conclusion that the Experiment 1 finding of impairment at lag-1 reflected an impact on perceptual processes rather than a more global impact. When targets followed a negative distractor, performance at lag-1 was significantly poorer than at lag-8 and poorer than when following a neutral distractor.

## Experiment 3

While Experiment 1 revealed that performance impairments following negative distractors were comparable at lag-1 and lag-2, and Experiment 2 verified that such impairments were not due a global impact on response mechanisms, it is unclear if these two earliest lags represent the largest impact of emotional distractors. It is possible, for example, that the extraction of emotional information unfolds slowly enough that emotional distractors wield their biggest impact at even later lags (e.g., lags 3 or 4). Electrophysiological evidence may suggest such as a delay, as key neural signatures responsive to emotional stimuli onset only after about 150-ms post-presentation [31–32]. In order to probe whether emotion-induced blindness decreases linearly with increasing lag, Experiment 3 tested target accuracy across the first four serial positions following the distractor. If temporal proximity to a distractor contributes to emotion-induced blindness, there should be a recovery from impairment with increased lag and no dip in performance at lags 3 or 4.

Following from the observation that about half the participants in Experiment 1 showed a trend towards better performance at lag-1 than at lag-2 (despite the fact that no evidence had emerged for lag-1-sparing at the group level), Experiment 3 also introduced self-report measures of attentional control, anxiety, and rumination in an exploratory bid to probe potentially systematic differences in lag-1 performance. These three measures were chosen for the following reasons: a) Attentional control might aid in overcoming emotional distractors, with participants high in attentional control better at ignoring distractors in order to focus on task-relevant information. To the degree that individual differences in lag-1 performance reflect fundamental mechanisms of attention, it might also be expected that self-rated attentional control would predict such differences instead of affect-related measures. b) Anxiety and rumination might be associated with an increased rate of extraction of negative emotional information from images. Rumination (particularly a “brooding” subscale) refers to the tendency to dwell on negative experiences from the past and is often associated with depression [43–44], and evidence suggests that rumination is associated with a biased attention toward negative stimuli [45–46].

### Method

#### Participants

Twenty-one undergraduates from the University of Delaware participated in Experiment 2 for course credit (mean age = 19.2 years; 12 female, 9 male). Sample size was driven by appropriateness for the primary comparison of performance at lags 1 and 2 relative to lags 3 and 4, not for the probing of individual differences, as this latter aspect was exploratory. All participants provided written informed consent as approved by the University of Delaware’s Human Subjects Review Board, Approval Number 136021–4.

#### Materials and Procedure

The same general procedure was used in Experiment 3 as in Experiments 1 and 2. Participants searched for a single rotated target in a stream of images. The distractor could occur at serial position 4 or 6, and critically, the distractor and target could be separated by 1, 2, 3, or 4 serial positions (lag-1 through lag-4). With more conditions than in the previous experiments, Experiment 2 included 384 trials presented over 4 blocks. Participants also completed three questionnaires after completing the emotion-induced blindness task, including the Attentional Control (AC) Scale [47], the Spielberger State-Trait Anxiety Inventory (STAI-T; as a measure of anxiety) [48], and the Ruminative Responses Scale [49], which includes subscales for reflection and brooding. Reflection and brooding both factor into an overarching index of rumination, with reflection referring to contemplation as a way to cope, whereas brooding refers more to “moody pondering” [49].

### Results

**Fig 3:**
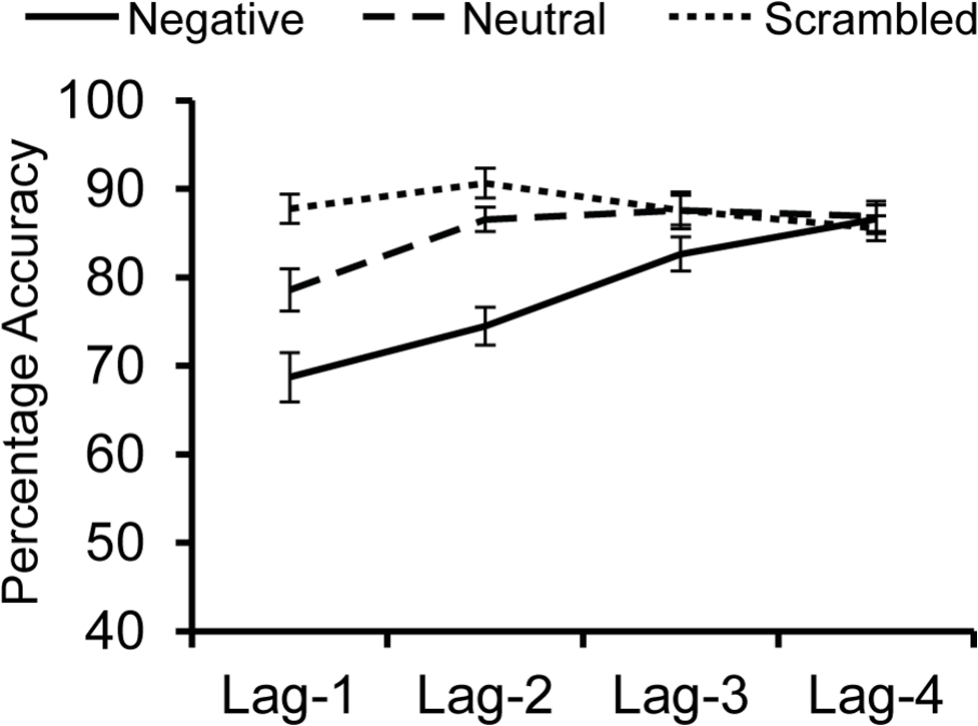
Average percent accuracies for target detection in Experiment 3. The time course of target detection when presented one image (lag-1), two images (lag-2), three images (lag-3), or four images (lag-4) after negative, neutral, or scrambled distractors.

An overall 3 (Distractor Type: negative, neutral, scrambled) x 4 (lag-1 vs. lag-2 vs. lag-3 vs. lag-4) within-subjects ANOVA revealed a significant main effect of Distractor Type, *F*(2, 40) = 33.935, *p* < .001, η_p_^2^ = .629, a significant main effect of Lag, *F*(3, 60) = 15.461, *p* < .001, η_p_^2^ = .436, and a significant Distractor Type X Lag interaction, *F*(6, 120) = 10.283, *p* < .001, η_p_^2^ = .340 (see Fig 3).

As in Experiment 1, accuracy for targets in streams with negative distractors was significantly impaired at both lag-1 and lag-2 compared to accuracy in streams with neutral and scrambled distractors (*t*s>3.4, *p*s<.003). In contrast, accuracy for targets after negative distractors did not differ from streams with neutral or scrambled distractors at lag-3 or lag-4 (*t*s<2, *p*s>.05), although the difference in performance at lag-3 between negative and scrambled distractors was “marginally” significant, *t*(20) = 1.947, *p* = .066, *d*_*z*_ = .425. A subsequent repeated-measures ANOVA on performance following negative distractors revealed a significant effect of lag, *F*(3, 60) = 19.731, *p*<.001, η_p_^2^ = .497, with the largest impairment at lag-1. A linear contrast analysis on these trials suggested a significant linear increase in performance between lags 1 and 4, *F*(1, 20) = 92.717, *p*<.001 η_p_^2^ = .823, suggesting a strong, linear trend between lag-1 and lag-4.

To explore individual differences in lag-1 performance following emotional distractors, we subtracted performance at lag-1 from lag-2 in the negative distractor condition. We found that there was a significant positive correlation between this difference score and brooding, *r* = 0.483, *p* = .036. No other individual difference measures significantly correlated with the difference in performance between lag-1 and lag-2, except for a marginally significantly correlation between the difference score and rumination broadly, *R* = 0.445, *p* = .056, consistent with brooding as a factor feeding into the rumination index.

**Fig 4:**
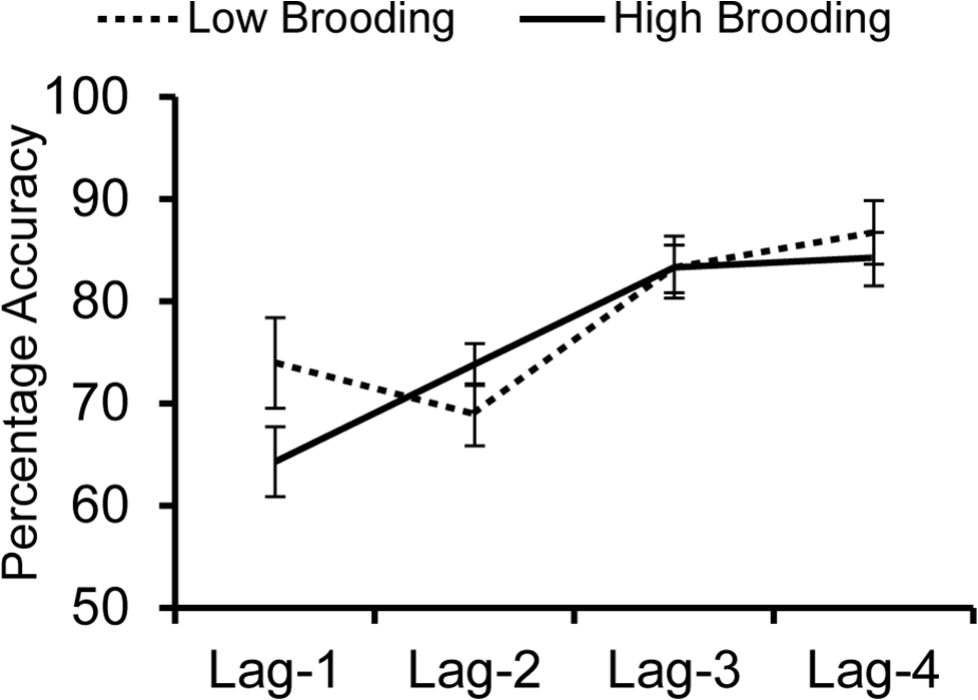
Average percent accuracies of participants with low vs. high levels of brooding (based on a median split), in streams with negative distractors.

To better understand the relationship between brooding and the relative impact of emotional distractors at lags 1 and 2, we split participants based on the median of brooding scores (see Fig 4). Three participants with scores that fell on the median were excluded from these analyses. A 2 (lag-1 vs. lag-2) X 2 (high vs. low brooding) mixed ANOVA on trials with emotional distractors revealed no significant main effect of Lag, *F*(1, 14) = 0.590, *p* = .455, η_p_^2^ = .040 and no significant main effect of Brooding, *F*(1, 14) = 0.366, *p* = .556, η_p_^2^ = .025; however, a significant interaction between lag and brooding did emerge, *F*(1, 14) = 6.420, *p* = .024, η_p_^2^ = .314. Participants who measured high in brooding performed marginally better at lag-2 (*M* = 74.0%, *SD* = 9.1%) than at lag-1 (*M* = 64.3%, *SD* = 13.2%), *t*(8) = 2.030, *p* = .077, *d*_*z*_ = .677, whereas participants who measured low in brooding performed marginally worse at lag-2 (*M* = 68.9%, *SD* = 4.9%) than lag-1 (*M* = 74.0%, *SD* = 8.2%), *t*(6) = 2.086, *p* = .082, *d*_*z*_ = .789. It is worth noting that while some of these results fell within the realm of “marginal” significance, our sample size was chosen on the basis of the primary hypothesis of a linear change in performance across lags, not for testing individual differences. Although these individual differences results must be regarded as exploratory, they are suggestive that differences associated with negative affect determine the early temporal pattern of disruption from emotional distractors.

### Discussion

In addition to probing the time-course of emotion-induced blindness, Experiment 3 provided converging support for findings of impairment in target detection at lag-1 in emotion-induced blindness, which was at least as robust as the impairments at later lags. Intriguingly, with the inclusion of measures to probe potentially systematic individual differences, Experiment 3 also provided preliminary evidence that the relative impact of emotional distractors at lag-1 vs. lag-2 may be subject to individual differences, with some people (e.g., high brooders) exhibiting a faster onset of emotion-induced blindness. The relation between brooding and performance at lag-1 may be most consistent with the notion that what appears to be lag-1-sparing in low brooders stems from a later onset of competition between targets and emotional distractors rather than individual differences in fundamental mechanisms of rapid attention. Although this pattern might appropriately be regarded as merely suggestive pending further verification, it raises the possibility that individuals characterized by high negative affect tend to more rapidly extract negative emotional information from visual stimuli. While beyond the scope of this study, additional work is also necessary to determine whether such a pattern extends only to negatively valenced stimuli or to emotional information generally.

## General Discussion

In Experiment 1, emotional stimuli similarly impaired perception of targets presented one (lag-1) or two (lag-2) positions later, while Experiment 2 revealed that accuracy significantly improved by lag-8 compared to lag-1. In Experiment 3, the impact of emotional distractors was tested across four successive serial positions, and accuracy following emotionally negative distractors, at a group level, improved linearly from lag-1 to lag-4. The three experiments converged to demonstrate that the extraction of emotional information from complex visual scenes occurs so rapidly as to disrupt perception of targets that appear in the same location only 100-ms later (i.e., lag-1).

Intriguingly, some participants exhibited better target perception at lag-1 than at lag-2 following an emotional distractor. In Experiment 1, roughly half of the participants trended in this direction, whereas the other half trended in the reverse direction. However, in Experiment 1 no additional measures were taken that might otherwise have allowed us to probe for a systematic reason for such individual differences. In Experiment 3, we administered self-report measures of attentional control and negative affect in an exploratory bid to probe for such systematic differences. The resulting data suggested that individuals characterized by relatively high levels of negative affect, as indexed by a brooding ruminative style, might experience a more rapid onset of emotion-induced blindness: participants scoring higher in this measure performed worse following emotional distractors when the target appeared at lag-1 than at lag-2, whereas those scoring low in this measure experienced a maximum impact of emotional distractors when the target appeared at lag-2. Future work is necessary not only to verify this pattern, but also to test whether such differences emerge purely as a function of the temporal distance between targets and distractors or are instead tied to the number of items that separate them. As noted in the Discussion for Experiment 3, further work is also needed in order to determine of such differences generalize beyond negative emotional stimuli.

The absence of robust lag-1-sparing when collapsed across participants stands in contrast to common findings from the attentional blink literature, where perception of the second of two targets remains intact when it appears immediately after the first target (e.g., [7,21–23]). This contrast is consistent with suggestions that although emotion-induced blindness and the attentional blink appear phenomenally related, there may be mechanistic distinctions between them. For example, the AB appears to stem from late-stage, central bottlenecks [7,21,25], whereas EIB may stem from spatiotemporally driven competition between targets and emotional distractors [6,17]. This suggestion has emerged from findings that whereas the AB typically appears to affect the entire visual field ([18], but see [50]), EIB appears to be spatially localized, occurring only at the location of the emotional distractor (see [51]). According to this account, EIB occurs when the emotional distractor and the subsequent target compete for dominance under conditions where they appear to be ambiguously linked to an overlapping point in time and space [29]. If so, then target perception impairments in EIB would be expected to increase as the temporal distance between target and emotional distractor decreases.

Competition accounts have also been proposed for the AB [52–56]. Evidence for this competition comes from reversals in lag-1 sparing, such that participants often confuse the order of T1 and T2 [52] and sometimes integrate T1 and T2 into a single percept at lag-1 [52–54]. Recent theories of AB argue that when both T1 and T2 are presented close together, they can compete with one another for representation when processed in the same “episode” [53,55,56]. At this point, it is unclear if the competition processes implicated in the AB are the same or similar to those being proposed in EIB. Future research should continue to explore the similarities of the competition mechanisms proposed for these two phenomena.

It is important to note that although the present findings are consistent with the notion that emotion-induced blindness does not show the common AB pattern of lag-1 sparing, they do not necessitate the conclusion that EIB necessarily reflects different mechanisms than those that drive the AB. For example, work within the more typical, dual-target, non-emotional attentional blink literature suggests that the robustness of lag-1-sparing can be modulated by the nature of the stimulus that appears at lag-1: letters within a stream seem to be associated with more lag-1-sparing than pictures of objects within a stream [57]. Thus, it is conceivable that the relative weakness of lag-1-sparing at a group level in the present experiments stems from the complexity of the pictorial stimuli rather than the operation of mechanisms distinct from those involved in the attentional blink.

Although preliminary, the fact that spared performance at lag-1 in EIB was predicted by trait negative affect in the current studies may go some way toward informing interpretation of our overall results. This relationship may be more consistent with a slower extraction of negative emotional information among people characterized by low negative affect–and therefore delayed onset of competition between targets and distractors–than with differences in the fundamental attentional mechanisms implicated in the attentional blink. While greater negative affect has been associated with a greater AB magnitude [35–39], it has not been found to modulate lag-1 sparing in the AB [37,39].

In sum, the present experiments probed the temporal properties of emotion-induced blindness, specifically focusing on its onset. Our data revealed that, generally, emotional distractors can impair the perception of subsequent targets presented just after them (lag-1), but that there are individual differences in the onset of disruption. While the sample size in Experiment 3 was modest, the apparent presence of systematic individual differences was informative, suggesting that negative emotional distractors begin to compete with targets sooner among people characterized by high negative affect. Larger scale follow-up investigations could provide further insight into individual differences in the rapidity of emotion-induced perceptual disruption, which may be of clinical significance.
